# Prevalence of major depressive disorder in Iranian men from 2011 to 2022; a systematic review and meta-analysis

**DOI:** 10.1186/s12888-025-06616-7

**Published:** 2025-03-03

**Authors:** Ahmad Hajebi, Ilia Zamani Hajiabadi, Afsaneh Fendereski, Robabeh Ghodssighassemabadi, Arshia Zamani Hajiabadi, Jalil Hosseini, Keshvar Samadaee Gelehkolaee

**Affiliations:** 1 Research Center for Addiction & Risky Behaviors (ReCARB), Psychosocial Health Research Institute, Iran University of Medical Sciences, Tehran, Iran; 2 Department of Psychiatry, Faculty of Medicine, Iran University of Medical Sciences, Tehran, Iran; 3 Student research committee, School of Medicine, Semnan University of Medical Sciences, Semnan, Iran; 4 Department of biostatistics, School of Health, Mazandaran University of Medical Sciences, Sari, Iran; 5 Office of Biostatistics and Epidemiology, Université Paris- Saclay, Gustave Roussy, U1018, Inserm, Villejuif, 948 05 France; 6 Student research committee, School of Medicine, Mazandaran University of Medical Sciences, Sari, Iran; 7 Men’s Health and Reproductive Health Research Center, Shahid Beheshti University of Medical Sciences, Tehran, Iran; 8 Sexual and Reproductive Health Research Center, Department of Reproductive Health and Midwifery, Faculty of Nursing and Midwifery, Mazandaran University of Medical Sciences, Sari, Iran

**Keywords:** Major depressive disorder, Prevalence, Men, Iran, A systematic review

## Abstract

**Background:**

Major depressive disorder (MDD) is one of the health problems that imposes a large disease burden on the community. Its prevalence is based on gender. Therefore, this study aims to investigate the prevalence of MDD in Iranian men between 2011 and 2022.

**Methods:**

The Medline, Embase, Scopus, Web of Sciences, PsycINfo, and Iranian databases were searched for studies published from January 2011 to December 2022. Two independent reviewers screened the observational studies conducted on men aged 15 years and older with MDD. The interested outcomes were the prevalence type of MDD.

**Results:**

Data of 5 studies were included in the present meta-analysis. Among 10,667 participants in the study, 1479 (13.9%) individuals had a history of MDD. In the subgroup analysis, the average lifetime prevalence was 7.6% (95% confidence interval [CI]; 4.7 − 12.0%) and the point prevalence was 19.1% (95% confidence interval [CI]; 6.7 − 43.5%) based on random effect model. The 12-month prevalence was 10.8% (95% confidence interval [CI]; 9.8 − 11.9%) based on the single study.

**Conclusion:**

Preventive and therapeutic programs are strongly recommended due to the high prevalence of major depression in Iranian men and the lack of sufficient studies in this field.

**Supplementary Information:**

The online version contains supplementary material available at 10.1186/s12888-025-06616-7.

## Introduction

Major depressive disorder (MDD) is the most common mood disorder worldwide which affects a large number of the general population. It can cause serious social, economic, professional, and personal problems in individual life [1]. The Diagnostic and Statistical Manual of Mental Disorders, Fifth Edition, Text Revision (DSM-5-TR), outlines specific criteria for MDD diagnosis. A diagnosis requires the presence of at least five symptoms during a period of twoweeks, representing a change from previous functioning, with at least one symptom being either a depressed mood or a loss of interest or pleasure. The main symptoms are depressed mood, markedly diminished interest or pleasure, significant weight loss, insomnia or hypersomnia, psychomotor agitation or retardation, fatigue or loss of energy, feelings of worthlessness, diminished ability to think or concentrate, and recurrent thoughts of death [2]. The global burden of MDD in men increased between 1990 and 2019, the age-standardized DALY rates increased from 352 in 1990 to 354 in 2019 [3]. During MDD, a person may experience suicidal ideation [4]. A systematic review conducted by Dong et al. (2019) on individuals aged 15 years and older reported that the lifetime pooled prevalence of suicide attempts among MDD patients is 31%, 1-year prevalence is 8%, and 1-month prevalence is 24% [5]. Additionally, another systematic review conducted by Hong et al. (2021) on individuals aged 15 years and older reported that in MDD patients compared to non-MDD controls, the odds ratios (ORs) are higher for various types of lifetime suicidality such as suicidal ideation (SI: 2.88), suicide plan (SP: 9.51), and suicide attempt (SA: 3.45) [4].

Many factors affect the prevalence of MDD such as age, gender, culture, vocation, social and economic status, physical health status [6]. The lifetime prevalence of MDD ranged from 2 to 21% in different countries [6]. The results of a systematic review of data from 30 countries showed that the prevalence rates of MDD were 10.8% for a lifetime, 7.2% for 12-months, and 12.9% for a point prevalence and a higher prevalence was observed among Asian compared to the other countries [7].

Iran is an Asian country with a population of about 80 million people from diverse racial backgrounds and cultures. The prevalence of MDD has been reported in various studies with different results. The result of a systematic review in 2010 showed that the lifetime prevalence of MDD in Iran was 4.1% [8]. However, studies from 2011 onward revealed different prevalence. Vandad Sharifi et al. reported that the one-year prevalence of MDD was 12.7% in the general population of Iran, with females (17.3%) having a higher prevalence than males (11.9%) [9]. In a study conducted in Kermanshah city, Iran, it was found that 8.3% of the total sample had MDD, with a higher prevalence among females [10]. A cluster analysis of the psychiatric profile of Iranian youth in Ravansar city, Iran, MDD prevalence was 21.6% (from a total number of 2991 samples) and disability was reported among 92.8% of MDD cases [11]. During the COVID-19 pandemic, the prevalence of depression was 22.1%, with females and young adults being more affected [12]. From 2011 to 2022, several studies have been conducted on the prevalence of MDD in Iran, resulting in various reports. The current study focused on men and MDD diagnostic tools, meaning that studies that used MDD diagnostic tools rather than just screening were included. Men are less likely to seek help for mental health issues due to societal stigma, cultural norms, and gender expectations. This underreporting may lead to an underestimation of the true prevalence of MDD in men [13]. While women have a higher prevalence of MDD, men have a higher suicide rate, often linked to untreated or undiagnosed depression. Understanding the prevalence and unique presentation of MDD in men could help address this disparity and develop targeted suicide prevention strategies [14]. On the other hand, societal expectations often discourage men from expressing vulnerability, leading to a “silent epidemic” of mental health issues. Investigating MDD prevalence in men can shed light on the effects of these norms and highlight the need for gender-sensitive interventions [15]. Therefore, this study aims to investigate the prevalence of MDD in Iranian men between 2011 and 2022.

## Material and method

### Study design and search strategy

This study adhered to the Preferred Reporting Items for Systematic Reviews and Meta-Analyze (PRISMA) [16]. An extensive search for articles published between January 2011 and December 2022 was conducted across multiple databases including Medline, Web of Science, Scopus, Embase, PsycINFO, SID, Magiran, and Irandoc. In order to increase the search sensitivity in PubMed, we used Medical Subject Headings (MeSH) terms for extraction the keywords and equivalents and access all the relevant articles. The search strategy was designed in four steps (Table 1). For Embase database we used Emtree terms to extractkeywords and their equivalents. For other databases we used MeSH terms to extract keywords and equivalent with its specific search strategy which ismentioned in supplementary Table 1. Searching for grey literature was conducted on Google, Google Scholar, and the dissertation section of the ProQuest database.

**Table 1:** Search strategy in Medline database

Database	Search terms
MEDLINE (PubMed)	1. “Depressive Disorder“[mh] OR “Depressive Disorder, Major“[mh] OR “Depression“[mh] OR “Mental Disorders“[mh] OR “Mood Disorders“[mh] OR “Psychiatry and Psychology Category“[mh] OR “Dysthymic Disorder“[mh] OR Major Depressive Disorders [tiab] OR Psychoses, Involutional[tiab] OR Involutional Depression[tiab] OR Involutional Psychoses[tiab] OR Depressive Neuroses[tiab] OR Depressive Syndrome[tiab] OR Endogenous Depressions[tiab] OR Neurotic Depressions[tiab] OR Unipolar Depression[tiab] OR Affective Disorders[tiab] OR Psychiatric Illnesses[tiab] OR Psychiatric Diseases[tiab] OR Mental Illnesses[tiab] OR Behavior Disorders[tiab] OR Behavioral Symptom[tiab] OR Psychiatric Diagnosis[tiab] OR Severe Mental Disorders[tiab] 2. “Incidence“[mh] OR “Epidemiologic Methods“[mh] OR “Epidemiologic Measurements“[mh] OR “Population Characteristics“[mh] OR “Epidemiology“[mh] OR Incidence Proportions[tiab] OR Incidence Rates[tiab] OR epidemics[tiab] OR frequency[tiab] OR surveillance[tiab] OR occurrence[tiab] OR outbreaks[tiab] OR prevalence[tiab] OR incidence[tiab] 3. “Iran“[mh] OR Islamic Republic of Iran[tiab] 4. # 1AND #2 AND #3.

### Data collection, inclusion and exclusion criteria

We included all studies with the prevalence of depressive disorders conducted on men aged 15 years and older during the years 2011 to 2022 in Iran. The main outcome in this research is the prevalence of major depression disorders, which was measured using standard assessment tools, including SCID, CIDI, PHQ9, and MDQ, which are among the most commonly used tools for MDD diagnosis. All extracted studies were imported into the EndNote software, and duplicate titles were identified and excluded. All studies were independently evaluated by two reviewers in severalsteps. The reviewers were selected from two experts in the field of medical sciences (AZH and IZH). In the first stepof review, the titles and abstracts of the studies were assessed for their relevance to the research topic (prevalence of depression), and if they were relevant, the full text of the studies were extracted, and irrelevant studies were excluded. Furthermore, if systematic review studies were found at this stage, their references were manually searched, and if they hadnot been included among the registered references in the EndNote software, they were added to the references, and their titles and abstracts were assessed relevancy. In the second step, after reading the full text of the articles and based on the selection criteria (inclusion and exclusion criteria), the data of these studies were then summarized in a checklist designed based on PRISMA statement guidelines [16]. The extracted data are summarized in Table 2. In case of disagreement between the reviewers at any of the above stages, the issue was resolved through discussion and, if necessary, the presence of a third reviewer (a psychiatric specialist AH). All studies that met the following criteria were included in the review:

1. The full text of the article was available in the specified databases.
2. The study was of appropriate quality.
3. If multiple studies were found to have been conducted in the same location and time, the study with the largest sample size was selected, and the other studies were excluded.
4. The study mentioned the prevalence of major depression disorders in men.
5. The studies were conducted among the Iranian population.
6. The study was either cross-sectional or cohort in nature.

**Fig. 1:**
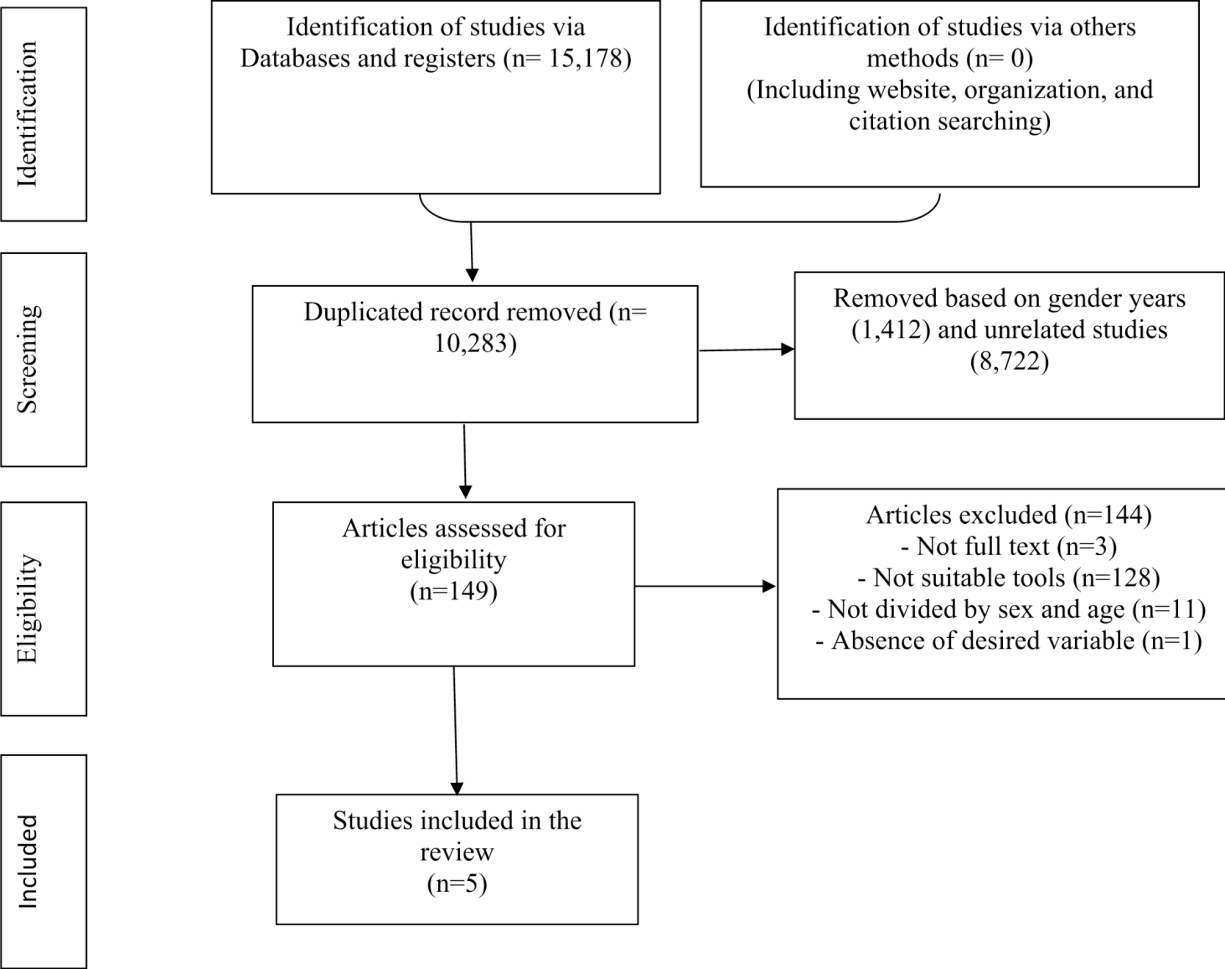
PRISMA flow diagram of the present study. MDD: Major depresive disorder

Criteria for study exclusion:

1. Studies that did not report the prevalence of MDD.
2. Studies in which the prevalence of MDD had not been reported separately by gender.
3. Studies whose full text was not accessible.
4. Studies conducted on men younger than 15 years old (Fig.1).

**Table 2:** Main characteristics of the included studies

First Author	Publication Year	Study design	Study Population Number/Men	Study Population-	Sampling method	Age distribution	Diagnostic tool	Prevalence* LT 12- MP PP
Maryam Shirzadi [10]	2019	cross-sectional	2102/1098	General Population	Phase1: a randomly selected cluster sampling Phase2: a positive result were randomly	20–71/33.8(12.6)	Interviewed by psychologists and DSM-IV (SCID, life time)&(GHQ-28)	65(5.9%) LT
Habibolah Khazaie [11]	2018	cross-sectional	2991/1328	The adult PERSIAN Cohort	Census	15–34/27.02 ± 5.06 (both)	interviews were performed by two psychologists & CIDI	646 (21.6) both, (9.6) male LT
Reza Shahriarirad [23]	2021	cross-sectional web-based survey	8591/2888	General population	Convenience sampling method	15–87/34.37 ± 11.25 (both)	PHQ-9	(15.1%)both PP
Vandad Sharifi [9]	2015	cross-sectional	7886/3387	General population	A three-stage cluster sampling	15–64	psychologist interviewer (CIDI, version 2.1)	10.2 (8.9–11.4) 12-MP
Saman Maroufizadeh [12]	2022	cross-sectional	5328/1966	General population	Convenience sampling method	> 18,32.95 (10.53)both	PHQ-9	(30.1%) PP

*Type of Prevalence: LT: Lifetime, 12-MP: 12-Month prevalence, PP: Point prevalence

### Quality assessment

Quality assessment of studies was conducted using the Checklist for Prevalence Studies tool prepared by the Joanna Briggs Institute (JBI) [17]. This checklist includes nine items: appropriateness of the selected population, appropriateness of the sampling method, sample size adequacy, detailed description of study individuals and methods, adequacy of the selected sample, use of appropriate method to identify research subjects, use of appropriate method or tool for outcome assessment, use of appropriate statistical methods, and appropriate response rate. Articles with a score of less than or equal to 3 are of low quality, 4–6 are of moderate quality, and 7–9 are of high quality (Table 3). The agreement between the two reviewers was assessed using the kappa coefficient. In this study kappa coefficient was 96%.

**Table 3:** Risk of bias assessment of included studies according to Checklist for Prevalence studies tool prepared by the Joanna Briggs Institute (JBI)

Studies										
	1	2	3	4	5	6	7	8	9	Total Score
Maryam Shirzadi	Yes	Yes	Unclear	Yes	Yes	Yes	Yes	Yes	Yes	8
Habibolah Khazaie	Yes	Yes	Yes	Yes	Yes	Yes	Yes	Yes	Yes	9
Reza Shahriarirad	Yes	Yes	Yes	Unclear	Yes	Yes	Yes	Yes	Yes	8
Vandad Sharifi	Yes	Yes	Yes	Yes	Yes	Yes	Yes	Yes	Yes	9
Saman Maroufizadeh	Yes	Unclear	Not applicable	Unclear	Yes	Unclear	Yes	Yes	Yes	5

### Statistical analysis

Data analyses were performed using R software (Version 4.3.2). The package “meta” was used for the calculation of an overall proportion and for plotting of all the figures. The function “metaprop” was used to estimate the summary effect in the random-effects model with 95% confidence intervals (CI). We assigned weights to each study based on their sample sizes for calculating the overall estimate, ensuring that larger studies contributed more to the overall findings. The τ2 and I2 statistics were used to assess between-study heterogeneity. A significant *τ*^*2*^ suggests statistically significant between-study variation and therefore moderators should be explored. The *I*^*2*^ statistic represents the percentage of total variation across studies with a value of 75% or greater corresponding to high heterogeneity [18]. Considering that the studies examined three different types of prevalence, including point prevalence, lifetime prevalence, and 12-month prevalence, subgroup analysis was conducted to investigate each type of prevalence. A forest plot was made to represent the overall prevalence of depression and depression within subgroups. Due to the small number of studies, publication bias and sensitivity analysis to assess the robustness of the findings were not performed. This addition clarifies how sample size was utilized in calculating the overall or pooled estimate, addressing the reviewer’s comment effectively.

## Results

A summary of the five studies included in this analysis is presented in Table 2, which includes the authors of the studies, publication years, sample size, percentage of men, diagnostic method, age range, type of prevalence (Point prevalence, 12-month prevalence or Life Time prevalence), and the number of participants diagnosed with MDD. Samples sizes ranged from 1098 to 3387, aged 15–87 years. Depression was assessed using standardized self-report questionnaires in all the studies. Among3 studies diagnostic interviews were employed in addition to a standardized self-report questionnaire.

### MDD prevalence estimate

Among 10,667 participants in the study, 1,479 individuals (13.9%) reported a history of Major Depressive Disorder. We have summarized these findings in the subgroup analysis.

### Subgroup analysis

**Fig. 2:**
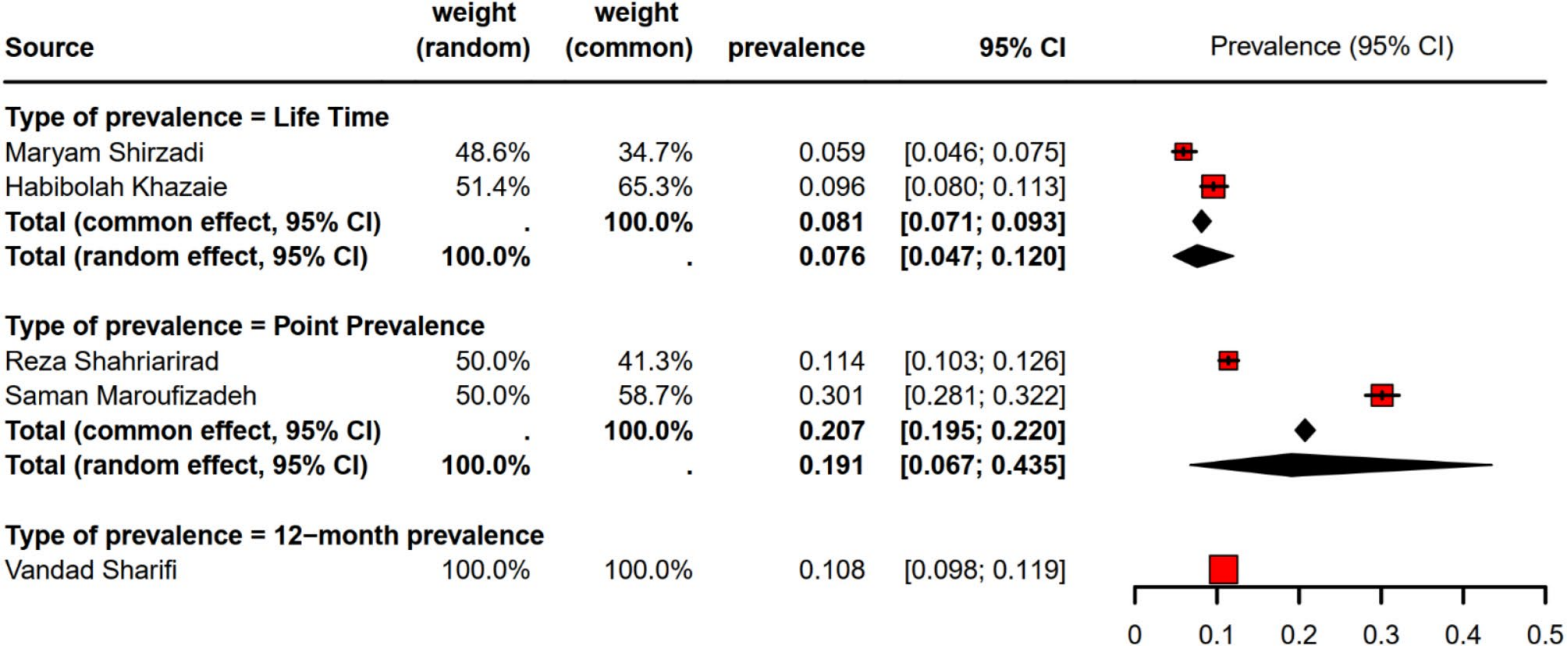
Forest plot for MDD prevalence categorized by diferent type of study

There were 3 types of prevalence, including point prevalence with two studies, 12-month prevalence with one study, and lifetime prevalence with two studies. The average lifetime prevalence was 7.6% (95% confidence interval [CI]; 4.7 − 12.0%) and the point prevalence was 19.1% (95% confidence interval [CI]; 6.7 − 43.5%) based on random effect model. The 12-month prevalence was 10.8% (95% confidence interval [CI]; 9.8 − 11.9%) based on the single study of Sharifi et al. A high level of heterogeneity was observed in lifetime (I^2^ = 90.7%, *τ*^*2*^ = 0.122, *p* = 0.001) and point prevalence (I^2^ = 99.6%, *τ*^*2*^ = 0.728, *p* < 0.001). Figure 2 shows the mean prevalence of all types according to the random effect and the fixed effect model.

## Discussion

Estimating the prevalence of mental disorders, especially major depression, is crucial due to the high disease burden in society and its importance for policymaking in the health system [3]. Therefore, the present study was conducted to investigate the prevalence of major depressive disorder in Iranian men between 2011 until 2022. The literature reports three types of MDD (including lifetime, 12-month prevalence, and point prevalence), each of which can be investigated from a specialized point of view. It should be noted that the present study focused on studies that used diagnostic tools for major depressive disorder, while studies using other tools that only reported screening were excluded from the final analysis As a result, a limited number of studies were included in the final analysis.

According to the results of the present study, the lifetime, 12-month, and point prevalence of MDD in Iranian men are 7.6%, 10.8%, and 19.1%, respectively. A systematic review study done by Lim et al., in a community of 30 countries reported that MDD has a lifetime prevalence of 10.8%, 12-month prevalence of 7.2% and point prevalence of 12.9% in which Asian countries have higher prevalence than the others [7]. An interesting finding in this study is that diagnoses made using interview-based assessment tools had a lower prevalence (8.5%) compared to diagnoses made using self-reporting tools (17.3%). This report highlights the importance of considering diagnostic tools in the studies discussed in the present study. Additionally, a subset of included studies of the present study were conducted during the COVID-19 pandemic, which could lead to significant differences in its results compared to other studies.

Gharraee et al., (2019) reported the prevalence of MDD in the general population [1]. The results of their study showed that the point prevalence of MDD in the community was 4.1% and 2.3% in men. The present study investigated a different period (2011 to 2022) and used different databases and diagnostic tools to diagnose MDD. This study also reported high heterogeneity among the included studies, similar to the present study. The study’s findings align with evidence-based research indicating a growing public health concern [19, 20].

A meta-analysis study was conducted by Salari et al. in 2020 to investigate severe depression in the Iranian adult community (over 50 years old). The results showed that the prevalence of depression in people over 50 years old in Iranian society was 8.3% [21]. The study differed from ours in terms of the type of depression, target population, and diagnostic tools used.

In 2021, Jaafari et al. investigated the prevalence of depression in Iranian students with an average age of 21 years. The results of their study showed that the prevalence of depression in boys was 51.3% [22]. The databases examined in this study were in line with the present study. The target group, depression measurement tool, and the type of depression investigated are the reasons for the difference in the results of the two studies.

A study conducted in 2020 by Gutiérrez et al. found that MDD has a lifetime prevalence of 2–21% and a 12-month prevalence of 1.1–10.4%.In this study, the prevalence of major depression in European countries was higher than in Asian countries [6]. Contrary to the Grace Y. Lim study that reported Asian countries have a higher prevalence of MDD than the others [7]. Given the high rates of suicide attempts and successful suicides in communities, this issue poses a significant health problem. There is an increasing need to investigate the causes and interventions that can help reduce suicides.

### Strengths and limitations

One of this study’s strengths is its diagnostic tools for study selection and its comprehensive and systematic approach to searching for relevant studies. The high heterogeneity among studies and the lack of descriptive studies in some provinces made it impossible to conduct a meta-analysis with high accuracy.

## Conclusion

The current meta-analysis revealed a noticeable lifetime, 12-month prevalence, and point prevalence of major depressive disorder in Iranian men. It also showed that using the appropriate tool for diagnostic measures can make a significant difference in the results. The present study also includes some of the studies conducted during the COVID-19 pandemic, which can show the prevalence of MDD in the outbreak, especially the point prevalence. Considering these results and the high burden of this disorder for communities, the need to design and implement preventive and treatment programs is more and more necessary than ever. Additionally, valid studies in this field are not available in many provinces and this study highlights the necessity of conducting descriptive prevalence studies in the provinces.

## Electronic supplementary material

Below is the link to the electronic supplementary material.

**Supplementary Material 1**: **Supplementary Table 1**: Search strategy in others databases

## Data Availability

No datasets were generated or analysed during the current study.

## References

[1] Gharraee B, Tajrishi KZ, Sheybani F, Tahmasbi N, Mirzaei M, Farahani H, et al. Prevalence of major depressive disorder in the general population of Iran: a systematic review and meta-analysis. Med J Islamic Repub Iran. 2019;33:151. 10.34171/mjiri.33.151 PMC7137832 32280657

[2] Edition F. Diagnostic and statistical manual of mental disorders. Am Psychiatric Assoc. 2013;21(21):591–643.

[3] Li S, Xu Y, Zheng L, Pang H, Zhang Q, Lou L, et al. Sex difference in global burden of major depressive disorder: findings from the global burden of disease study 2019. Front Psychiatry. 2022;13:789305. 35264985 10.3389/fpsyt.2022.789305 PMC8898927

[4] Cai H, Xie X-M, Zhang Q, Cui X, Lin J-X, Sim K, et al. Prevalence of suicidality in major depressive disorder: a systematic review and meta-analysis of comparative studies. Front Psychiatry. 2021;12:690130. 34603096 10.3389/fpsyt.2021.690130 PMC8481605

[5] Dong M, Zeng L-N, Lu L, Li X-H, Ungvari GS, Ng CH, et al. Prevalence of suicide attempt in individuals with major depressive disorder: a meta-analysis of observational surveys. Psychol Med. 2019;49(10):1691–704. 30178722 10.1017/S0033291718002301

[6] Gutiérrez-Rojas L, Porras-Segovia A, Dunne H, Andrade-González N, Cervilla JA. Prevalence and correlates of major depressive disorder: a systematic review. Brazilian J Psychiatry. 2020;42:657–72. 10.1590/1516-4446-2019-0650 PMC7678895 32756809

[7] Lim GY, Tam WW, Lu Y, Ho CS, Zhang MW, Ho RC. Prevalence of depression in the community from 30 countries between 1994 and 2014. Sci Rep. 2018;8(1):2861. 29434331 10.1038/s41598-018-21243-x PMC5809481

[8] Sadeghirad B, Haghdoost A-A, Amin-Esmaeili M, Ananloo ES, Ghaeli P, Rahimi-Movaghar A, et al. Epidemiology of major depressive disorder in Iran: a systematic review and meta-analysis. Int J Prev Med. 2010;1(2):81. 21566767 PMC3075476

[9] Sharifi V, Amin-Esmaeili M, Hajebi A, Motevalian A, Radgoodarzi R, Hefazi M, et al. Twelve-month prevalence and correlates of psychiatric disorders in Iran: the Iranian Mental Health Survey, 2011. Arch Iran Med. 2015;18(2):0. 25644794

[10] Shirzadi M, Jozanifard Y, Eskandari S, Farhang S, Khazaei H. An epidemiological survey of psychiatric disorders in Iran: Kermanshah. Asian J Psychiatry. 2019;43:67–9. 10.1016/j.ajp.2019.04.004 31096141

[11] Khazaie H, Najafi F, Hamzeh B, Chehri A, Rahimi-Movaghar A, Amin-Esmaeili M, et al. Cluster analysis of psychiatric profile, its correlates, and using mental health services among the young people aged 15–34: findings from the first phase of Iranian youth cohort in Ravansar. Soc Psychiatry Psychiatr Epidemiol. 2018;53:1339–48. 30145626 10.1007/s00127-018-1580-4

[12] Maroufizadeh S, Pourshaikhian M, Pourramzani A, Sheikholeslami F, Moghadamnia MT, Alavi SA. Prevalence of anxiety and depression in the Iranian General Population during the COVID-19 pandemic: a web-based cross-sectional study. Iran J Psychiatry. 2022;17(2):230. 36262765 10.21203/rs.3.rs-39082/v1 PMC9533345

[13] Cole BP, Davidson MM. Exploring men’s perceptions about male depression. Psychol Men Masculinities. 2019;20(4):459.

[14] Sher L. Suicide in men: an underappreciated public health challenge. Eur Arch Psychiatry Clin NeuroSci. 2020;270(2):277–8. 31286193 10.1007/s00406-019-01041-w

[15] Ziegenhagen MM. An E-Course & men’s Development Cohort to address the issue of isolation and disconnection experienced by Modern men: a Holistic & Integrative Approach. Fuller Theological Seminary, School of Psychology; 2024.

[16] Moher D, Liberati A, Tetzlaff J, Altman DG. PRISMA Group* t. Preferred reporting items for systematic reviews and meta-analyses: the PRISMA statement. Ann Intern Med. 2009;151(4):264–9. 19622511 10.7326/0003-4819-151-4-200908180-00135

[17] Munn Z, Moola S, Lisy K, Riitano D, Tufanaru C. Methodological guidance for systematic reviews of observational epidemiological studies reporting prevalence and cumulative incidence data. JBI Evid Implement. 2015;13(3):147–53. 10.1097/XEB.0000000000000054 26317388

[18] Higgins JP, Thompson SG, Deeks JJ, Altman DG. Measuring inconsistency in meta-analyses. BMJ. 2003;327(7414):557–60. 12958120 10.1136/bmj.327.7414.557 PMC192859

[19] Husky MM, Léon C, du Roscoät E, Vasiliadis H-M. Prevalence of past-year major depressive episode among young adults between 2005 and 2021: results from four national representative surveys in France. J Affect Disord. 2023;342:192–200. 37730150 10.1016/j.jad.2023.09.019

[20] Ten Have M, Tuithof M, van Dorsselaer S, Schouten F, Luik AI, de Graaf R. Prevalence and trends of common mental disorders from 2007-2009 to 2019‐2022: results from the Netherlands Mental Health Survey and Incidence studies (NEMESIS), including comparison of prevalence rates before vs. during the COVID‐19 pandemic. World Psychiatry. 2023;22(2):275–85. 37159351 10.1002/wps.21087 PMC10168151

[21] Salari N, Mohammadi M, Vaisi-Raygani A, Abdi A, Shohaimi S, Khaledipaveh B, et al. The prevalence of severe depression in Iranian older adult: a meta-analysis and meta-regression. BMC Geriatr. 2020;20:1–8. 10.1186/s12877-020-1444-0 PMC6998325 32013895

[22] Jaafari Z, Farhadi A, Lari FA, Mousavi FS, Moltafet H, Dashti E et al. Prevalence of depression in Iranian college students: a systematic review and meta-analysis. Iran J Psychiatry Behav Sci. 2021;15(1).

[23] Shahriarirad R, Erfani A, Ranjbar K, Bazrafshan A, Mirahmadizadeh A. The mental health impact of COVID-19 outbreak: a Nationwide Survey in Iran. Int J Mental Health Syst. 2021;15:1–13. 10.1186/s13033-021-00445-3 PMC7913044 33640006

