# Supplementary material for: Prevalence of major depressive disorder in Iranian men from 2011 to 2022; a systematic review and meta-analysis
Source: BMC Psychiatry. 2025 Mar 3;25:189. doi: 10.1186/s12888-025-06616-7 (PMC11874079; doi:10.1186/s12888-025-06616-7)
Supplement: Supplementary file 1 — Supplementary Material 1: Supplementary Table 1: Search strategy in others databases [file 12888_2025_6616_MOESM1_ESM.docx]

Supplementary Table 1: Search strategy in others databases

| Database | Search terms |
| --- | --- |
| Embase: | **1.** 'major depression'/exp OR 'Major Depression Inventory'/exp OR 'depression assessment'/exp OR 'mental disease assessment'/exp OR 'mood disorder assessment'/exp OR 'depression'/exp OR 'mood disorder'/exp OR 'mental disease '/exp OR ' major depressive disorder**’:ab,ti OR ‘**major depressive episode’:ab,ti OR ‘depressive disease’:ab,ti OR ‘depressive disorder’:ab,ti OR ‘depressive episode ’:ab,ti OR ‘depressive illness ’:ab,ti OR ‘depressive personality disorder’:ab,ti OR ‘depressive state ’:ab,ti OR ‘depressive symptom ’:ab,ti OR ‘depressive syndrome’:ab,ti OR ‘mental depression’:ab,ti OR ‘parental depression’:ab,ti OR ‘unipolar depression’:ab,ti OR ‘unipolar disorder ’:ab,ti OR ‘psychiatric disorder assessment ’:ab,ti OR ‘Major Depression Inventory (MDI)’:ab,ti OR ‘affective disorders’:ab,ti OR ‘ affective disturbance ’:ab,ti OR ‘affective illness ’:ab,ti OR ‘mood disorders’:ab,ti OR ‘mood disturbances’:ab,ti OR ‘abnormal mental state’:ab,ti OR ‘mental change’:ab,ti OR ‘mental confusion’:ab,ti OR ‘mental defect’:ab,ti OR ‘mental abnormality’:ab,ti OR ‘diseased mental state’:ab,ti OR ‘disturbed mental state’:ab,ti OR ‘neurodevelopmental disorders’:ab,ti OR ‘psychiatric disease ’:ab,ti OR ‘psychiatric disorder’:ab,ti OR ‘psychiatric illness’:ab,ti  **2.** 'Prevalence'/exp OR 'prevalence ratio'/exp OR 'prevalence changes'/exp OR 'Epidemiology'/exp OR 'incidence '/exp OR 'Frequency'/exp OR 'incidence rate’:ab,ti OR ‘rate’:ab,ti OR ‘incidence’:ab,ti OR ‘prevalence study’:ab,ti OR ‘prevalence ratio’:ab,ti OR ‘epidemiology’:ab,ti OR ‘epidemiologic survey’:ab,ti  **3.** 'Male'/exp OR 'boy'/exp OR ‘males’:ab,ti OR ‘man’:ab,ti OR ‘men’:ab,ti  **4.** 'Iran'/exp OR 'Iranian'/exp OR ‘Iran’:ab,ti OR ‘Persia’:ab,ti  **5.** # 1AND #2 AND #3 AND #4. |
| Scopus: | 1.TITEL-ABS-KEY(“major depressive disorder“ OR “major depressive episode“ OR “depressive disease“ OR “depressive disorder“ OR “depressive episode“ OR “depressive illness“ OR “depressive personality disorder“ OR “depressive state“ OR “depressive symptom“ OR “depressive syndrome“ OR “mental depression“ OR “parental depression“ OR “unipolar depression“ OR “unipolar disorder“ OR “psychiatric disorder assessment“ OR “Major Depression Inventory“ OR “affective disorders“ OR “affective disturbance“ OR “affective illness“ OR “mood disorders“ OR “mood disturbances“ OR “abnormal mental state“ OR “mental change“ OR “mental confusion“ OR “mental defect“ OR “mental abnormality“ OR “diseased mental state“ OR “disturbed mental state“ OR “neurodevelopmental disorders“ OR “psychiatric disease“ OR “psychiatric disorder“ OR “psychiatric illness“ OR “incidence rate“)  2. TITEL-ABS-KEY (“rate“ OR “incidence“ OR “prevalence study“ OR “prevalence ratio“ OR “epidemiology“ OR “epidemiologic survey“)  3. TITEL-ABS-KEY(“Iran“ OR “Persia“)  4. TITEL-ABS-KEY(“males“ OR “man“ OR “men“)  5. # 1AND #2 AND #3 AND #4. |
| Web of science | 1.TS= (“major depressive disorder“ OR “major depressive episode“ OR “depressive disease“ OR “depressive disorder“ OR “depressive episode“ OR “depressive illness“ OR “depressive personality disorder“ OR “depressive state“ OR “depressive symptom“ OR “depressive syndrome“ OR “mental depression“ OR “parental depression“ OR “unipolar depression“ OR “unipolar disorder“ OR “psychiatric disorder assessment“ OR “Major Depression Inventory“ OR “affective disorders“ OR “affective disturbance“ OR “affective illness“ OR “mood disorders“ OR “mood disturbances“ OR “abnormal mental state“ OR “mental change“ OR “mental confusion“ OR “mental defect“ OR “mental abnormality“ OR “diseased mental state“ OR “disturbed mental state“ OR “neurodevelopmental disorders“ OR “psychiatric disease“ OR “psychiatric disorder“ OR “psychiatric illness“ OR “incidence rate“)  2. TS= (“rate“ OR “incidence“ OR “prevalence study“ OR “prevalence ratio“ OR “epidemiology“ OR “epidemiologic survey“)  3. TS= (“Iran“ OR “Persia“)  4. TS= (“males“ OR “man“ OR “men“)  5. # 1AND #2 AND #3 AND #4. |
